# Prevalence of Medication Non-Adherence and Associated Factors among Diabetic Patients in A Tertiary Hospital at Debre Markos, Northwest Ethiopia

**DOI:** 10.4314/ejhs.v32i4.12

**Published:** 2022-07

**Authors:** Yihunie Mitiku, Anteneh Belayneh, Bantayehu Addis Tegegne, Bekalu Kebede, Dehnnet Abebe, Yalemgeta Biyazin, Bereket Bahiru, Abtie Abebaw, Hylemariam Mihiretie Mengist, Melese Getachew

**Affiliations:** 1 School of Medicine, Debre Markos University, Debre Markos, Ethiopia; 2 Department of Pharmacy, College of Health Sciences, Debre Markos University, Debre Markos, Ethiopia; 3 Department of Pediatrics and Child Health Nursing, College of Health Sciences, Debre Markos University, Debre Markos, Ethiopia; 4 Department of Pharmacy, College of Health Sciences, Bahir Dar University, Bahir Dar, Ethiopia; 5 Department of Medical Laboratory Sciences, College of Health Sciences, Debre Markos University, Debre Markos, Ethiopia

**Keywords:** Medication, Non-adherence, Diabetes, Debre Markos, Ethiopia

## Abstract

**Background:**

Non-adherence to prescribed medications is possibly the most common reason for poor treatment outcomes among people with diabetes although its rate is highly variable. Data on the magnitude of medication non-adherence and associated factors are scarce in the study area. This study aimed to assess the rate of non-adherence and associated factors among diabetic patients at Debre Markos Comprehensive Specialized Hospital.

**Methods:**

A cross-sectional study was conducted from June 17 to July 17, 2021. Study participants were selected using a simple random sampling technique. Data were collected with a pre-tested structured questionnaire and entered into SPSS version 25. Logistic regression was utilized to determine predictors of medication non-adherence at a significance level of ≤ 0.05.

**Results:**

A total of 176 study participants were enrolled in the study. About 59% of the study participants had type-2 diabetes mellitus. The prevalence of non-adherence to anti-diabetic medications was found to be 41.5%. Male sex, rural residence, being divorced, being merchant, self- or family-borne medical cost, and presence of comorbidities were significantly associated with increased rate of non-adherence to anti-diabetic medications.

**Conclusion:**

The prevalence of non-adherence to medications among diabetic patients is significantly high in the study area. Public health measures should be strengthened to decrease nonadherence among diabetic patients.

## Introduction

Diabetes is a chronic metabolic disease characterized by elevated blood glucose levels, and it is a major health problem with a growing prevalence. It is recognized by the World Health Organization (WHO) as one of the world's most important public health problems for prevention, diagnosis, and treatment along with increasing obesity and cardiovascular diseases. It is estimated that about 422 million people have diabetes globally. Most of these diabetic patients are citizens of low-and middle-income countries. About 1.5 million annual deaths are directly attributed to diabetes (1,2). This chronic disease requires a life-long treatment that greatly increases the risk of serious, long-term complications. Offering long-term monitoring and treatment in case of diabetes is not easy for the healthcare systems in sub-Saharan Africa where more focus is given to managing acute infections (3). Although the current prevalence of diabetes mellitus in the region is lower than in developed countries, it is an alarming health problem (4,5). A recently published meta-analysis study indicated that the prevalence of diabetes mellitus in Ethiopia is estimated to be 6.5% with a varying proportion among regions (6).

WHO defines adherence to long-term therapy as “the extent to which a person's behavior; taking medications, following a diet, and/or executing lifestyle changes, corresponds with agreed recommendations from a healthcare provider” (7). Medication non-adherence comprehends a wide range of behaviors whether they are encountered deliberately or not leading to either underuse or overuse of medications prescribed. Underutilization of medications is manifested by a delay or failure to fill prescriptions, splitting pills, skipping doses, stopping medication early, and not refilling a prescription (8). Poor metabolic control is usually associated with non-adherence to antidiabetic medications. Both acute and long-term complications might then be encountered due to poor metabolic control (7,9).

Studies showed that there is unsatisfactory adherence to anti-diabetic medications and glycemic self-control in type 2 diabetic patients globally (10). Non-adherence to medications is possibly the most common reason for poor health outcomes among people with diabetes. The safety and effectiveness of anti-diabetic medications are compromised due to poor adherence of patients which leads to a higher rate of morbidity and mortality. This also contributes to elevated costs of the health care system either directly or indirectly.

Highly variable rates of non-adherence to anti-diabetic medications are reported; thus, its impacts on treatment outcome should be considered. It is noted that many diabetic patients take less than the prescribed amounts of their medications. The level of non-adherence to medications among diabetes patients is variable in Ethiopia ranging from 34% (11) to 68.8% (12). Diabetic related complications (13,14), sex (13), age (15), level of education (13,16), lower income (15), cost of transport to the hospital (11,17), longer duration under treatment (15,18,19), history of admission (17,20), and taking alcohol (11) were reported to be predictors of medication non-adherence. Improving adherence to diabetes treatment thus is a vital public health issue. Age, knowledge about medication, and the presence of co-morbidities are among the most commonly reported determinants of medication non-adherence to diabetic treatment (9,21). Determining the level of non-adherence to medications and associated factors among diabetic patients helps to design more appropriate interventions. No study is documented about the rate of medication non-adherence, associated factors, and its impacts on treatment outcomes among diabetes patients in the study area. The aim of this study was, therefore, to assess medication non-adherence and associated factors among diabetic patients at Debre Markos Comprehensive Specialized Hospital (DMCSH), Debre Markos town, North West Ethiopia.

## Materials and Methods

**Study setting, design, and period**: An institution-based cross-sectional study was conducted at DMCSH from June 17 to July 17, 2021. The hospital is found in Debre Markos town which is located in the northwestern part of Ethiopia 299 km away from the capital, Addis Ababa. It was established in 1965 G.C which currently services a total of about 3.5 million people coming from East Gojjam, West Gojjam, and Awi zones of the Amhara region, and some parts of the Oromia region. It provides service in four major clinical departments; internal medicine, surgery, pediatric and gynecology/obstetric, and other clinical departments such as dentistry, psychiatry, orthopedics, diabetes, dermatology, and reproductive health services. During the study period, a total of 1800 ambulatory diabetes patients were attending the diabetic clinic on a regular follow-up basis.

**Study population**: All type 1 and type 2 diabetes patients attending the diabetes clinic of DMCSH were considered as source populations while all diabetic patients receiving any of the ant-diabetic medications were our study populations. Patients aged ≥ 18 years who were on follow-up for at least 6 months were included in the study whereas critically ill patients and patients with mental illness were excluded from the study as these patients can't provide information about their medication.

**Sampling size determination and sampling technique**: The sample size was determined by using a single population proportion formula by taking 34% proportion of non-adherence to anti-diabetic medications from a similar study conducted at Dilla University Referral Hospital, Ethiopia (11), a confidence level of 95%, and marginal error of 5%. The initial sample size was determined to be 176. Nonetheless, the sample size was adjusted as the source population was less than 10,000 by using the correction formula. Accordingly, the adjusted sample size was determined to be 161. By adding a 10% non-response rate, a total sample size of 178 was considered. Finally, the study participants were enrolled using a simple random sampling technique.

**Study variables**: Non-adherence to anti-diabetic medications was dependent variable of this study. The different independent variables used were socio-demographic factors (age, sex, marital status, residency, average monthly income, educational level, smoking, alcohol intake), factors related to regimen (number and type of drugs prescribed), and patient related factors (Diabetes-related complications, history of hospitalization, comorbidities, medical cost coverage).

**Data collection methods**: A structured questionnaire, developed by reviewing different works of literature, was used to collect data. It was first developed in English, translated into the local language (Amharic), and retranslated back to English to check for consistency. The questionnaire comprised items related to sociodemographic characteristics (age, sex, marital status, residency, average monthly income, educational status, and occupation), coverage of medical costs, current health complaints other than diabetes, and questions to assess the level of adherence to their medication. The questionnaire was pre-tested with 5% of the sample size a week before the actual data collection period in Finote Selam primary hospital and necessary adjustments were done accordingly. Data were collected by two trained nurses through face-to-face interview.

Daily check-up of the completed questionnaires was done to ensure data completeness. Besides, a checklist was used to abstract data from patients' medical charts. The data collected from patients' charts included type of diabetes mellitus, diabetes-related complications, comorbidities, type and number of medications prescribed, frequency of medication administration, and presence of side effects of medications.

**Data processing and analysis**: Four questions were included in the questionnaire to assess adherence to medication which are adopted from a validated adherence tool developed by Morisky et al. (1986). The questions were designed to assess patients' forgetfulness and carelessness about taking medications, and stopping medications when feeling better or worse. The response choices were “yes” or “no” which are rated as 1 and 0, respectively. Patients who scored 0 were considered highly adherent to their medication. Whereas, those who scored 1 or 2 were considered to have medium adherence, and those who scored 3 or 4 were considered to have low adherence levels. For statistical purposes, only respondents with high adherence were considered as adherent in this study whereas those with medium and low adherence were considered as non-adherent (2,22). Data were cleaned, coded, and entered into Statistical Package for Social Sciences (SPSS) version 25 software for analysis. Descriptive statistics such as frequencies and percentages were computed and results were presented in the form of tables to summarize categorical variables. Binary logistic regression models were utilized to determine the association of predictors with the outcome variable. Variables with a p-value of ≤ 0.25 at 95% CI in the bivariate logistic regression were further analyzed with multivariable logistic regression to control confounders. A p-value of ≤0.05 at 95% CI was considered statistically significant.

**Ethical approval**: The study was conducted after it was ethically approved by the Research and Ethical Review Committee of the School of Medicine (SN: S/R/C/58/03/21; Date: 17/06/2021), Debre Markos University. Then, a supporting letter was written to the administrators of DMCSH. Verbal informed consent was obtained from each participant after describing the objective of the study, procedures of selection, and assurance of confidentiality. All the information obtained from the study participants was coded to keep confidentiality. The principles of the Declaration of Helsinki (23) were strictly followed throughout the study.

## Results

**Socio-demographic characteristics**: A total of 176 study participants were enrolled in this study with a 98.9% response rate. About 52% (92/176) of them were females and about half of the study participants (51.7%) were rural residents. Most of the study participants (56.82%) fall in the age group of 31 to 60 years old. Concerning their marital status, most (64.77%) of them were married. About 62% of the study participants had at least completed primary education while 38.07% were unable to read and write. Farming (40.91%) and merchandising (25.57%) were the first and second most common occupations of study participants, respectively. However, around two-thirds (63.07%) of them had an average monthly income of less than 2000 Ethiopian Birr (ETB). Moreover, 19 (10.8%) were found to be alcohol consumers (Table 1).

**Table 1:** Socio-demographic characteristics of the study participants at DMCSH (n = 176) from June 17 to July 17, 2021

Variable		Adherent (Frequency, (%))	Non-Adherent (Frequency, (%))	Total (Frequency, (%))
**Age**	18–30	45 (72.58)	17 (27.42)	62 (35.230
	31–60	56 (56)	44 (44)	100 (56.82)
	>60	2 (14.29)	12 (85.71)	14 (7.95)
**Sex**	Male	37 (44.05)	47 (55.95)	84 (47.73)
	Female	66 (71.74)	26 (28.26)	92 (52.27)
Residence	Urban	42 (49.41)	43 (50.59)	85 (48.30)
	Rural	61 (67.03)	30 (32.97)	91 (51.70)
	Single	29 (70.73)	12 (29.27)	41 (23.30)
	Married	66 (57.90)	48 (42.10)	114 (64.77)
	Divorced	6 (35.30)	11 (64.70)	17 (9.66)
	Widowed	2 (50)	2 (50)	4 (2.27)
Educational status	No formal education	43 (64.18)	24 (35.82)	67 (38.07)
	Primary education	35 (53.03)	31 (46.97)	66 (37.5)
	Secondary education	6 (50)	6 (50)	12 (6.82)
	Diploma and above	19 (61.29)	12 (38.71)	31 (17.61)
Occupation	Civil servant	8 (5.33)	7 (46.67)	15 (8.52)
	Merchant	14 (31.11)	31 (68.89)	45 (25.57)
	Farmer	50 (69.44)	22 (30.56)	72 (40.91)
	Retired	3 (25)	9 (75)	12 (6.82)
	Other	28 (87.5)	4 (12.5)	32 (18.18)
Average monthly income (ETB)	<1000	36 (62.09)	22 (37.91)	58 (32.96)
	1000–2000	28 (52.83)	25 (47.17)	53 (30.11)
	>2000	39 (60)	26 (40)	65 (36.93)
Medical cost coverage	Government	87 (67.97)	41 (32.03)	128 (72.73)
	Self	7 (30.43)	16 (69.57)	23 (13.07)
	Family members	9 (42.86)	12 (57.14)	21 (11.93)
	Employer organization	0 (0)	4 (100)	4 (2.27)
Drinking alcohol	No	90 (57.32)	67 (42.68)	157 (89.20)
	Yes	13 (68.42)	6 (31.58)	19 (10.80)

**Clinical and medical characteristics**: As shown in Table 2, most (59.09%) of the study participants had type-2 diabetes mellitus. Moreover, approximately half (50.57%) of them have been taking anti-diabetic medications for less than five years. A smalld number (11.36%) of participants used to measure their blood sugar levels at home on a regular basis. Only 39 (22.16%) of the study participants were presented with one or more comorbidities. Hypertension (15.91%) was the most commonly reported comorbidity followed by chronic renal failure (2.84%). Seventy-three (41.48%) of them had a previous history of admission of which 81.82% were due to diabetes.

**Table 2:** Clinical and medical characteristics of study participants DMCSH (n = 176) from June 17 to July 17, 2021

Variable		Adherent Frequency (%)	Non-Adherent Frequency (%)	Total Frequency (%)
Type of Diabetes	Type 1	51 (70.83)	21 (29.17)	72 (40.91)
	Type 2	52 (50)	52 (50)	104 (59.09)
Duration of illness	<5 years	60 (67.42)	29 (32.58)	89 (50.57)
	5–10 years	30 (53.57)	26 (46.43)	56 (31.82)
	>10 years	13 (41.94)	18 (58.06)	31 (17.61)
Presence of comorbidity	No	90 (65.69)	47 (34.31)	137 (77.84)
	Yes	13 (33.33)	26 (66.67)	39 (22.16)
Type of comorbidity	Heart failure	2 (100)	0 (0)	2 (1.14)
	Neuropathy	0 (0)	2 (100)	2 (1.14)
	Hypertension	10 (35.71)	18 (64.29)	28 (15.91)
	Nephropathy	0 (0)	2 (100)	2 (1.14)
	Chronic renal failure	1 (20)	4 (80)	5 (2.84)
Previous history of admission	No	85 (82.52)	18 (17.48)	103 (58.52)
	Yes	62 (84.93)	11 (15.07)	73 (41.48)
Reason for hospitalizations	Diabetes	85 (56.25)	59 (43.75)	144 (81.82)
	Other	18 (56.25)	14 (43.75)	32 (18.18)
Regular blood sugar measurement	No	85 (54.49)	61 (45.51)	156 (88.64)
	Yes	8 (40)	12 (60)	20 (11.36)
Number and type of medications	Glibenclamide	0 (0)	2 (100)	2 (1.14)
	Metformin	15 (45.45)	18 (54.55)	33 (18.75)
	Insulin	75 (67.57)	36 (32.43)	111 (63.07)
	Glibenclamide + Metformin	11 (42.31)	15 (57.69)	26 (14.77)
	Insulin + OHA	2 (50)	2 (50)	4 (2.27)
Frequency of medication administration	Once per day	5 (50)	5 (50)	10 (5.68)
	≥ Twice a day	98 (59.04)	68 (41.96)	166 (94.32)
Current health complaints	None	84 (64.62)	46 (35.38)	130 (73.86)
	Heart problems	8 (33.33)	13 (66.67)	24 (13.64)
	Limb paralysis	2 (50)	2 (50)	4 (2.27)
	Visual impairment	3 (33.33)	6 (66.67)	9 (5.11)
	Renal problems	0	5 (100)	5 (2.84)
	Others	6 (85.71)	1 (14.29)	7 (3.98)
History of diabetes related complications	No	69 (70.41)	29 (29.59)	98 (55.68)
	Yes	34 (43.59)	44 (56.41)	78 (44.32)
Missing of doses within the last two weeks	No	98 (71.53)	39 (28.47)	137 (77.84)
	Yes	4 (10.53)	34 (89.47)	38 (22.16)
Reasons for missing doses (n = 38)	Feeling better	0 (0)	2 (100)	2 (1.14)
	Forgetting	2 (8.70)	21 (91.30)	23 (13.07)
	Fear of side effects	0 (0)	5 (100)	5 (2.84)
	Others	2 (25)	6 (75)	8 (4.55)

Regarding the number of medications prescribed, more than three-fourth (82.95%) of them were on mono-therapy where insulin was the most prescribed medication followed by metformin. Glibenclamide plus Metformin was the most commonly used combination therapy. More than three-fourth (77.84%) of the respondents reported that they did not miss any dose within the past two weeks. Forgetting to take medication was the most commonly reported reason for missing doses. Although a significant proportion (41.48%) of subjects had a history of admission due to diabetes mellitus, more than half (55.68%) of them had no diabetes-related complications.

**Figure 1:**
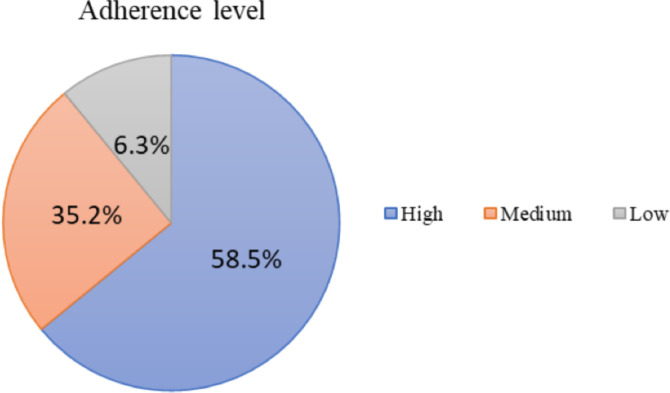
Adherence level of diabetic patients to their medications at DMCSH from June 17 to July 17, 2021.

**Non-adherence to anti-diabetic medications**: As depicted in Figure 1, 41.5% (95%CI [34.7–49.4]) of the respondents were non-adherent (35.2% medium and 6.3% low level of adherence) to their anti-diabetic medications.

The most prevalent reason for treatment non-adherence was forgetting (40.9%). Whereas, only 2 (1.1%) reported that they stop taking medications when they felt worse (Table 3). Half of the respondents with type-2 diabetes mellitus (DM) were non-adherent to their medications while 29.17% of type-1 DM patients were non-adherent. The highest and lowest extent of non-adherence were observed among respondents who were on anti-diabetic medications for more than ten years (58.06%) and less than five years (32.58%), respectively. Moreover, most patients with comorbidities (66.67%) and history of diabetes-related complications (56.41%) were found to be non-adherent (Table 2).

**Table 3:** Summary of diabetic patients' response to adherence related questions using Morisky tool, DMCSH from June 17 to July 17, 2021

Adherence related question	Yes n (%)]	No n (%)]
Do you ever forget to take your medication?	72 (40.91)	104 (59.09)
Are you sometimes careless at times about taking your medication?	15 (8.52)	161 (91.48)
When you feel better, do you sometimes stop taking your medication?	14 (7.95)	162 (92.05)
Sometimes if you feel worse when you take the medicine, do you stop taking it?	2 (1.14)	174 (98.86)

**Factors associated with non-adherence to anti-diabetic medications**: Older age, male sex, rural residence, being divorced, Type 2 DM, covering medical cost by self or family members, presence of comorbidity, and longer (>10 years) duration of treatment were found to be significantly associated with medication non-adherence in the bivariate analysis. After adjusting for confounders in multivariate analysis, being male, rural residence, being divorced, merchant occupation, covering the medical cost by self or family members, and presence of comorbidity were found to be significantly associated with non-adherence to anti-diabetic medication. Male patients were 6.47 times (AOR = 6.47, 95% CI: 2.37–17.68, P ≤ 0.001) more likely to be non-adherent than females while rural residents were 8.67 times (AOR = 8.67, 95% CI: 1.82–41.33, P = 0.007) non-adherent as compared with their counterparts. Similarly, being divorced increased the rate of medication non-adherence by 14.28 times (AOR = 14.28, 95% CI: 2.22–91.93, P = 0.005). Merchants were almost eight times (AOR = 7.99, 95% CI: 1.78–35.80, P = 0.007) more likely to be non-adherent to their anti-diabetic medications than civil servants. Compared to government-sponsored ones, patients whose medical cost was covered by themselves and family members were 4.34 (AOR = 4.34, 95% CI: 1.25–5.11, P = 0.021) and 5.58 (AOR = 5.58, 95% CI: 1.13–27.55, P = 0.035) times more likely to be non-adherent, respectively. Moreover, patients with one or more comorbidities were 3.87 times (AOR = 3.87, 95% CI: 1.31–11.40, P = 0.014) non-adherent compared to those who had no comorbidity (Table 4).

**Table 4:** Factors associated with non-adherence to medications among diabetic patients at DMCSH from June 17 to July 17, 2021

Variable		Bivariate		Multivariate	
		
		COR [95% CI1	P-value	AOR [95% CI1	P-value
Age	18–30	1			
	31–60	2.080(1.050–4.120)	0.036	-	0.429
	>60	15.882(3.214–78.47)	0.001	-	0.382
Sex	Male Female	3.225(1.725–6.028) 1	0.001	6.473 (2.369–17.683)	0.001
Residence	Urban	1			
	Rural	0.480 (0.261–0.884)	0.018	8.668(1.818–41.325)	0.007
Marital status	Single	1			
	Married	1.758(0.815–3.791)	0.150	1.595(0.444–5.733)	0.474
	Divorced	4.431 (1.333–14.723)	0.015	14.277(2.217–91.927)	0.005
	Widowed	2.417(0.304–19.194)	0.404	19.404(0.771–488.05)	0.072
Educational status					
No formal education		0.884(0.367–2.127)	0.783	-	-
Primary education		1.402(0.588–3.346)	0.446	-	-
Secondary education		1.583(0.413–6.063)	0.502	-	-
Diploma ' above		1			
Occupation					
Civil servant		1			
Merchant		2.531 (0.766–8.357)	0.128	7.988(1.782–35.804)	0.007
Farmer		0.503(0.162–1.559)	0.234	0.360(0.054–2.391)	0.290
Retired		3.429 (0.656–17.927)	0.144	1.970(0.141–27.430)	0.614
Other		0.163(0.038–0.702)	0.015	0.219(0.029–1.648)	0.140
Medical cost coverage					
Government		1			
Self		4.85(1.852–12.701)	0.001	4.339(1.246–15.108)	0.021
Family members		2.829(1.104–7.47)	0.030	5.581(1.131–27.554)	0.035
Employer organization		-	0.999	-	0.999
Type of diabetes mellitus	Type 1	1			
	Type 2	2.429(1.284–4.593)	0.006	-	0.334
Comorbidity	No	1			
	Yes	3.830(1.803–8.136)	0.001	3.871(1.314–11.404)	0.014
Previous history of	No	1			
admission	Yes	1.194(0.527–2.706)	0.672	-	-
Number of medications	One	1			
	≥ Two	2.102(0.949–4.656)	0.067	-	0.877
Duration of illness	<5 years	1			
	5–10 years	1.793(0.902–3.565)	0.096	-	0.451
	>10 years	2.865(1.237–6.635)	0.014		0.218

## Discussion

In this study, 104 (59.09%) and 72 (40.91%) of Type 2 and Type 1 DM patients were participated, respectively. The higher proportion of Type 1 DM patients enrolled in this study could be due to differences in appointment duration among the two groups. Type 2 DM patients attending at DMCSH are commonly appointed for two or more months because they are on oral hypoglycemic agents. On the other hand, Insulin is prescribed for Type 1 DM patients, which requires close follow-up and refrigeration, and hence they are usually appointed monthly. The proportion of non-adherence to medications among diabetic patients in different parts of the world is variable. In our study, we found that 73 (41.5%) of the respondents were non-adherent. The proportion of non-adherence to medications in our study was comparable with the finding of a similar study conducted in Zewditu Memorial Hospital, (16) and Gaza strip, Palestine (25).

The finding of this study was, however, higher than the findings of the studies conducted in Tikur Anbessa Specialized Hospital (Addis Ababa) (26), Assela General Hospital (27), Jimma University Specialized Hospital (28), Dilla University Referral Hospital (11), India (29), China (30), Denver metropolitan area (31), New York City (2), and South-West Germany (22). The possible reason for this might be due to differences in enrolled population respect to Types of DM patients, sample size, and socio-economic status.

In contrary to this, the proportion of non-adherence in our study was lower than findings of the studies conducted in Addis Ababa (4), Adigrat and Wukro General Hospitals (17), Kenya (20), Cameroon (19), India (32), Malaysia (33), Singapore (34), New York City (2), and USA (35). The impact of differences in sample size and adherence measurement tools on non-adherence levels should not be underestimated which could cause the differences observed among the results of the studies. The actual proportion of adherence to anti-diabetic medications may even be higher than the value obtained in our study as it was estimated based on patients' recall which usually overestimates patients' adherence levels (18). This is a worrying situation along with the ever-increasing prevalence of DM in developing countries, including Ethiopia (3). Moreover, the differences in the educational status of participants, residence, income, and level of awareness about medications could cause differences in the proportion of non-adherence as observed in different study settings.

Male patients were 6.47 times more likely to be non-adherent than females. A similar finding was reported by a study conducted in South-West Germany (22). This might be because males are usually engaged in outdoor activities in our country which may result in forgetting to take medications. Moreover, medical cost coverage might have contributed to this discrepancy as the medical cost of a higher proportion of female participants, compared with males, was covered by either government or employer organization (79.35% versus 65.5%).

Rural residents were 8.67 times non-adherent compared with urban residents. Patients living in rural areas probably face different challenges related to easy access to health facilities apart from information gaps. On the other hand, divorced individuals have 14.28 times increased rate of medication non-adherence compared to singles. The untoward psychosocial impact of divorce might have a detrimental effect on medication adherence due to lesser interest to take medications and forgetfulness (36).

Merchants were almost eight times more likely to be non-adherent than civil servants. This finding is supported by a report of a similar study conducted in Jimma town, Ethiopia. The increased rate of non-adherence to anti-diabetic medications among merchants may be attributed to the fact that they are busy and have multiple trips. Compared to those patients sponsored by the government, patients whose medical cost is covered by themselves and family members were 4.34 and 5.58 times more likely to be non-adherent, respectively. Now a days, the increasing cost of anti-diabetic medications has been entailed as a common challenge to improve medication adherence (37).

Moreover, patients who had comorbidities were 3.87 times non-adherent which is in agreement with a study done in Malaysia (33). This might be due to patients with two or more comorbidities will be challenged by polypharmacy, increased risk of developing side effects, and higher cost of medications.

The proportion of non-adherence to medications among DM patients is significantly high in the study area. Male sex, rural residence, being divorced, being a merchant, self- or family-borne medical cost, and presence of comorbidities were significantly associated with the increased rate of non-adherence to anti-diabetic medications. Strategies to increase the adherence rate of diabetic patients, including psychological and social support, proper counseling and improved interaction with healthcare professionals, support from family members, and addressing financial constraints to cover their medical costs should be implemented. In addition, multicenter prospective studies that minimize recall bias are recommended to decipher the real predictors of poor adherence.
